# Investigating the dynamic mechanism of user willingness to actively publish travel-related Electronic Word-of-Mouth (eWOM) on tourism platforms

**DOI:** 10.1371/journal.pone.0285773

**Published:** 2023-10-03

**Authors:** Silian Li, Fufan Liu

**Affiliations:** 1 Department of Design, Tongmyong University, Bushan, Republic of Korea; 2 Department of Innovation, Entrepreneurship and Information Systems, IESEG School of Management, Lille, France; Shandong Jiaotong University, CHINA

## Abstract

This study aims to uncover the dynamic mechanism of users’ willingness to actively publish Electronic word-of-Mouth (eWOM) on tourism platform. For this, a model with system dynamics and structural equation methods were constructed and validated. It was found that perceived usefulness, utilizing attitude, participatory, social identity, tourism experience, and platform agenda settings all had significant and positive effects on eWOM users’ willingness to actively publish eWOM on tourism platforms. However, perceived ease of use showed no effect. This study provides a reference paradigm for future studies on willingness to actively publish eWOM. The results bear implications for the management practices of tourism platforms operators and tourism destination operators. It also helps platform operators to develop relevant strategies for recovering the tourism industry in the post-COVID-19 era.

## Introduction

Tourism is gaining significant momentum in international communities [1–3]. With economic growth and transformations in consumer habits, the tourism industry has evolved from a niche to mass consumable in people’s pursuit of happiness and entertainment [1]. Tourism development is exceptionally dynamic faced with the colossal demand, and tourism products and services are rapidly upgrading [4]. Simultaneously, the impact of information technology on the social life manifested more clearly during the COVID-19 pandemic [1], as people are forced to rely on quick and easy access to new information in adapting to this new environment. Various online travel platforms and websites emerged, which provides online showcases for tourism products, services, and a massive amount of tourism information mainly in the form of eWOM [5,6]. Nowadays, eWOM has become an essential reference for tourists’ travel decisions [7] and means of reducing consumer decision-making risk [6,8]. Prior studies have shown that information provided by tourists who have experienced relevant tourism products and services is more up-to-date and credible than that provided by tourism agencies [9], which renders eWOM’s a non-negligible influence on tourists’ travel intention [6], destination choice [10], impression [11], trust [12], etc. In addition, the rapid development of different types of online tourism platforms is changing the way tourism eWOM is spread. On these platforms, tourists can both browse others’ eWOM reviews, interact with each other, and participate in eWOM distribution. Given the significant impact of eWOM, many scholars have dedicated to the study of tourism platform [13–15].

Based on the literature review, previous studies have focused on the impact of travel eWOM on tourism [8,16,17]. Few studies have focused on tourists’ willingness to publish eWOM actively [18]. However, increased content published by tourists on tourism platforms and their willingness to actively publish is crucial to marketing tourism destinations and tourism platforms. Thus, based on the summary of related tourism platform research literature [19–23], a potential research gap would be the lack of understanding of the dynamic mechanism behind tourists’ willingness to actively publish travel-related eWOM on tourism platforms. Previous studies have primarily focused on the impact of eWOM on tourism and have yet to dive into the specific factors that influence a tourist’s decision to actively publish eWOM [24]. This study aims to fill this gap by using a combination of system dynamics causality diagrams and technology acceptance models to measure and understand the dynamic mechanism behind tourists’ willingness to actively publish eWOM on tourism platforms. Additionally, as the literature review suggests that previous studies have used system dynamics and technology acceptance model separately to investigate willingness to publish eWOM [25], it is unclear how these two models interact and influence each other in our context. We use structural equation modeling to determine the path coefficients and the degree of influence among the dynamics factors. Overall, we empirically examine a model of the dynamics of users’ willingness to actively publish on tourism platforms through a carefully designed questionnaire, aiming to answer two key questions.

RQ1: How do the characteristics of tourism platforms affect users’ willingness to actively publish eWOM?
RQ2: What theoretical framework can be used to elucidate the underlying factors when investigating users’ willingness to actively publish eWOM on tourism platforms?

This study provides theoretical contribution by investigating the dynamic mechanism of user willingness to actively publish eWOM on tourism platforms by combining system dynamics causality diagrams and the technology acceptance model. With this approach, we contribute to existing literature on eWOM and tourism by providing a deeper understanding of the motivations and behaviors of users. In terms of practice, this study provides insights and recommendations for the development and management of tourism platforms, as well as the marketing strategies of tourism-related businesses. With better understanding of eWOM, platform developers and managers may create environments encouraging active participation in eWOM with complimentary features and incentives, as well as applying more efficienty marketing strategies aligned with the factors that influence user willingness and behavior.

## Theoretical background

### System dynamic*s*

System dynamics, founded by Forrester in 1958, is a discipline that analyzes and studies information feedback systems [26]. It is also a cross-cutting and broad discipline that strives to understand and solve system problems. Further, It is a tool to use systems thinking in order to solve dynamic and complex system problems based on systems science and computer simulation [27]. In terms of system methodology, system dynamics is a unification of the structural, functional, and historical approaches. System dynamics can reflect complex relationships between variables of different dimensions and is suitable for medium- and long-term prediction of dynamic and complex nonlinear systems [28]. It simulates the study of nonlinear, high-order, multivariate, multi-feedback system decision problems in a non-complete information state through system synthesis reasoning [26].

The complex and dynamic mechanism of users’ willingness to actively publish eWOM consists of tourism platforms, platform users (tourists), social relations, etc. Each part is interconnected and capable of influencing each other, while satisfying the modeling conditions of system dynamics. Thus, this study improves traditional modeling methods with the system dynamics modeling approach. The reason for choosing system dynamics is its potential in understanding the underlying mechanisms as well asl feedback loops that drive system behavior on top of just identifying those factors statically. In turn, this which would provide insights in how different factors interact and influence user behavior over time, thus providing insights on how the system can be manipulated to dynamically increase user engagement and participation. The feedback loop mechanism of system dynamics is studied from a system inquiry perspective. In this way, the behavioral patterns and characteristics of the system are derived. Furthermore, the causal diagram of the dynamics mechanism of users’ willingness to actively publish on the tourism platform is drawn.

### Technology acceptance model

Davis proposed the Technology Acceptance Model (TAM) in 1989 to study user acceptance of information systems using the Theory of Reasoned Action [29]. His original purpose of the TAM was to explain the determinants of widespread computer acceptance. Along technological progress and innovation, scholars have integrated the technology acceptance model with the Theory of Rational Behavior [30], Theory of Planned Behavior [31], Innovation Diffusion Theory [32], Use and Gratification Theory [33], etc. It has been continuously improved to adapt to the developmental changes of society. With social-wide application of big data and artificial intelligence, the characteristics of user groups could be more diversified and even qualitatively different, which implies new developments in TAM. With system dynamics principles and specific contextual considerations, out study further extends the TAM with the model of the dynamics mechanism of users’ willingness to actively publish eWOM on tourism platforms.

In the context of tourism platforms, TAM can be used to study user willingness to actively publish eWOM by understanding how the platform’s perceived usefulness and ease of use influence user behavior. For example, we may expect that if a tourism platform is perceived as useful for sharing travel experiences and recommendations as well as easy to use, these features may then increase users’ likelihood of actively publishing eWOM on that platform. Also, while understanding user’s static perception of the platform, the combination with dynamic mechanisms would shed light on how the factors in TAM interact and influence user behavior over time.

### Social identity theory

Henri Tajfel and John Turner developed Social Identity Theory (SIT) in the 1970s. This opened up a new field of study in social psychology and further explained the concept of intergroup relations [34]. Any kind of system is inseparable from the widespread recognition of social members. SIT suggests that social identity consists of three basic processes: categorization, identity, and comparison [35]. Categorization refers to people’s categorization of themselves into a community. Identity is the belief that one has the typical characteristics of that community members. Comparison is the evaluation of the community’s strengths and weaknesses, status, and reputation with which one identifies other communities. Through these three processes, people raise their values and self-esteem. SIT explains an individual’s identification and self-image perception of his or her group membership. In intergroup behavior, individual behavior is subject to the group categorization process. Individuals also need to perceive the value connotation of the social group. Thus, they regulate their behavior with the awareness of social categories and hope to obtain higher social recognition and prestige.

In the context of tourism platforms, social identity can shape an individual’s willingness to actively publish eWOM. For example, suppose an individual identifies with a "travel enthusiasts" group. In that case, they may be more likely to actively publish eWOM on tourism platforms to share their experiences and recommendations with others in their community. On the other hand, if an individual does not identify with a group of "travel enthusiasts", they may be less likely to actively publish eWOM.

Additionally, suppose an individual perceives the value connotation of the social group related to the tourism platform. In that case, they may regulate their behavior in alignment with the group, which will affect the willingness to actively publish eWOM. Social identity can also impact the type of eWOM an individual is willing to publish. For example, suppose an individual identifies with a group that values eco-tourism and sustainable travel. In that case, they may be more likely to actively publish eWOM highlighting the environmental and social sustainability of a certain destination or travel service. On the other hand, if an individual identifies with a group that values luxury and exclusivity, they may be more likely to actively publish eWOM that highlights high-end experiences and exclusive amenities.

Overall, social identity can shape an individual’s willingness to actively publish eWOM on tourism platforms by influencing the type of eWOM they are willing to publish and their motivation to share their experiences with others in their social group.

### Perceived usefulness

Perceived usefulness reflects the degree to which an individual subjectively perceives an increase in performance when using a particular system [29]. The prior study found that eWOM information usefulness positively affects attitudes toward eWOM information, which affects the forwarding of eWOM information [36]. Ghorbanzadeh and Saeednia [37] found that perceived usefulness affects Telegram users’ attitudes and consequently affects their delivery of positive eWOM when they conducted eWOM on Telegram users. The tourism platform has become a new social media platform in the mobile web era. Tourists can post destination-related content on the platform anytime and anywhere while interacting with a wide range of netizens. Tourists coudl receive everyone’s appreciation, recognition, and gratitude in different forms just by sharing their experiences, scenic photos, food recommendations, and other content during their travels. This increases the perceived self-efficacy of the tourist, which in turn increases the productivity of their subsequent work. Furthermore, the tourism platform provides tourists with video and photo production materials, including music, filters, video effects, and stickers. This provides an entertaining diversion for tourists at the end of a tiring day of travel. It allows them to relax, relieve their fatigue, ventilate and release their emotions, and thus increase their willingness to actively publish afterwards.

### Agenda setting theory

Max McCombs and Donald Shaw in 1972 proposed Agenda-Setting Theory [38]. This theory believes that mass media often cannot determine people’s specific views on a particular event or opinion. However, it can indirectly influence the thoughts of its audience by providing relevant information or setting relevant issues to influence people’s focus [38]. The Agenda Setting Theory inspires the mass media to construct relevant hot topics to attract the audience’s attention. This creates a new "pseudo-environment" [39] and establishes a consensus with the audience, thus influencing their relative perceptions.

In the case of tourism platforms, media can play a role in shaping the audience’s perception of travel destinations, experiences and services, which in turn can influence their willingness to actively publish eWOM on tourism platforms. For example, suppose the media consistently highlights positive experiences and reviews of a certain destination. In that case, it can create a sense of social validation among the audience, encouraging them to share their positive experiences through eWOM. On the other hand, if the media highlights negative experiences or issues related to a destination, it can discourage the audience from actively publishing eWOM.

It is important to note that not only the media that shape the audience’s perception but also other factors such as personal experience, word of mouth from family and friends, and overall sentiment in the society also play a role. However, the media can be important in creating a narrative around a certain topic and influencing the audience’s perception of it.

## Research hypothesis and model

### Perceived ease of use

Perceived ease of use reflects the extent to which individuals perceive that it saves them time and effort when using a particular system [29]. Mobile network technology improves, and mobile software performance stabilises, improving available services. Tourism platform introduces new features and new ways of interacting. The platform’s latest short video feature can facilitate video recording by tourists during the travel process. It enables them to restore the effects of the scenes that travel through the background music and special effects of the video, which can be genuinely and conveniently recorded and released on the platform later.

### Participatory

Participatory refers to how participants subjectively perceive that they are participating in an activity. In the era of we-media, user-generated content is no longer soley controlled and powered by "opinion leaders." Content production is gradually decentralized [40,41]. The decentralization of content production means that the content distribution on the Internet is no longer “monopolied” by selective professionals but jointly shaped by the participation and creation of all netizens. This, on the most surface level, gives the general public the opportunity and possibility to express themselves. Furthe for the tourists, the tourism platform’s promotion of the idea of "decentralization," gives them a certain level of participation [42] with their contents being potentially accessible to the public eye as well as gaining appreciation from others. All this will lead to a gradual change in visitors’ attitudes towards using the platform, which will inspire them to create more content on the platform relevant to their direct and future destinations.

### Social identity

Social identity refers to the connection between an individual and the group to which he or she belongs and the individual’s maintenance and identification with the group [43]. Individuals have a strong desire to be part of a group and to maintain close intra-group relationships with others. Some tourists strive to establish a feeling of organizational presence on the tourism platform by publishing travel-related material to earn the support and recognition of most netizens. This raises their image and brings attention on the platform, thus enhancing their social status with the possibility of becoming tourist “celebrities”. Prior study hinted that the psychodanamical drive of narcissism could be the most vital driver for users to use social media platforms, where one must strive to increase likes, shares, comments, etc., from other users with their posting of eWOM [44]. When fellow tourists browse travel reviews with popular hashtags to catch up with the trend, they may be influenced by the vibe and community culture of user-generated content. Gradually, they may follow suit and publish content on the tourism platform. Thus, tourists’ social interest in following online trends and integrating into popular online communities is constantly being met. As a result, they are identified as part of the platform.

### Platform agenda settings

Platform agenda settings refer to mass media’s efforts to set up popular topics or issues to influence users’ attention [45]. With big data, data mining and smart algorithms recommend "content that users may like.", which will keep more users in the "information cocoon." In other words, if users are interested in specific contents for a longer time, this will make them gradually generate creative ideas. When users are clueless about creation, the platform will supply them with inspiration by recommended personalized topics, which may fuel their willingness to create. Further, while the platform promotes and distributes information polular contents (e.g., hot issue, political debate, etc.) with affective potential, users may passively receive them and build emotional resonance. Users build up automated likeness by browsing the same hashtag topics and unconsciously accumulating similar video materials, thus creating the same topic. At a certain point, with enough accumulation and emotional charge, they are willing to participate and express themselves by creating related content [46].

### Tourism experience

Tourism experience refers to a tourist’s brief departure from the resident place to start a journey in the hope of gaining different experiences [47]. The tourism experience is a unique feeling generated by combining the tourist experience and the impression of the destination after exposure [48]. The tourism experience is defined as a collection of tourists’ conscious thoughts and feelings regarding the current tourism environment and a complex psychological, social, and cognitive interaction process [49]. It is also a feeling of physical and mental integration tourists get when they deeply integrate with the present situation of the tourist destination. The feeling may remain in memory as "unforgettable tourism experience" even long after the process [50]. The tourism experience of the destination is divided into positive and negative experiences [51]. A positive experience will make tourists actively publish some positive content related to tourism on the platform, such as travel tips, food recommendations, etc. Negative experience, on the other hand, will allow tourists to publish negative comments about the tourist destination on the platform, such as complaints and suggestions, bemoan, etc [52].

### Hypothesis development

**Fig 1:**
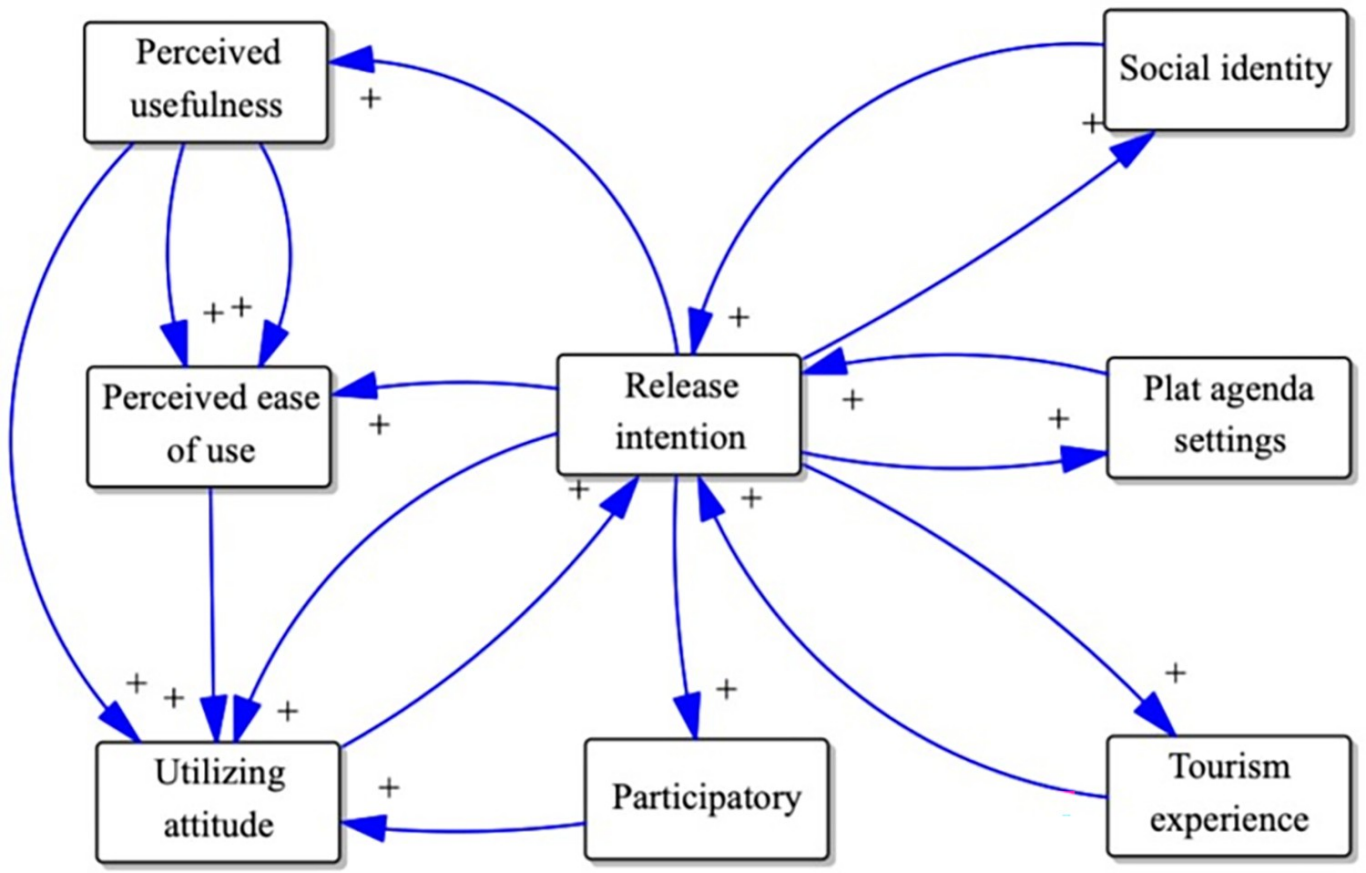
Users’ willingness to actively publish eWOM in causality diagram.

Based on system dynamics, social identity theory, and platform agenda setting theory, the causality diagram of users’ willingness to actively publish on the tourism platform was conceived by logical deduction. It is conceptually divided into three parts: tourism platform, platform users (tourists), and social relations, as shown in Fig 1. These constructs along with the related studies are presented in Table 1.

**Table 1:** Backgrounds of the research variables.

Author names	Abedi et al. [36]	Ghorbanzadeh & Saeednia [37]	Dutot [44]	Bi et al. [46]	Serra-Cantallops et al. [52]
Perceived ease of use		√			
Perceived usefulness	√	√			
Utilizing attitude	√	√			
Release intention	√	√			
Participatory		√			
Plat agenda settings				√	
Social identity			√		
Tourism experience					√

From Fig 1, there are seven causal feedback loops: First, perceived usefulness → + utilizing attitude → + release intention → + perceived usefulness. Second, perceived ease of use → + utilizing attitude → + release intention → + perceived ease of use. Third, participatory → + utilizing attitude → + release intention → + participatory. Fourth, perceived ease of use → + perceived usefulness → + perceived ease of use. Fifth, social identity → + release intention → + social identity. Sixth, plat agenda settings → + release intention → + plat agenda settings. Seventh, utilizing attitude → + release intention → + utilizing attitude. Eighth, tourism experience → + release intention → + tourism experience.

Therefore, this paper proposes.

H1: The perceived ease of use of the tourism platform positively influences users’ perceived usefulness.
H2: The Tourism platform’s perceived usefulness positively influences users’ utilizing attitude.
H3: The perceived ease of use of the tourism platform positively influences users’ utilizing attitudes.
H4: The utilizing attitude of tourism platforms positively influences users’ release intention.
H5: The participatory of the tourism platform positively influences users’ attitudes toward its use.
H6: The social identity generated by the tourism platform positively influences users’ release intention.
H7: The plat agenda settings of the tourism platform positively influence users’ release intention.
H8: Tourists’ tourism experience positively influences users’ release intention on the tourism platform.

### Research model

**Fig 2:**
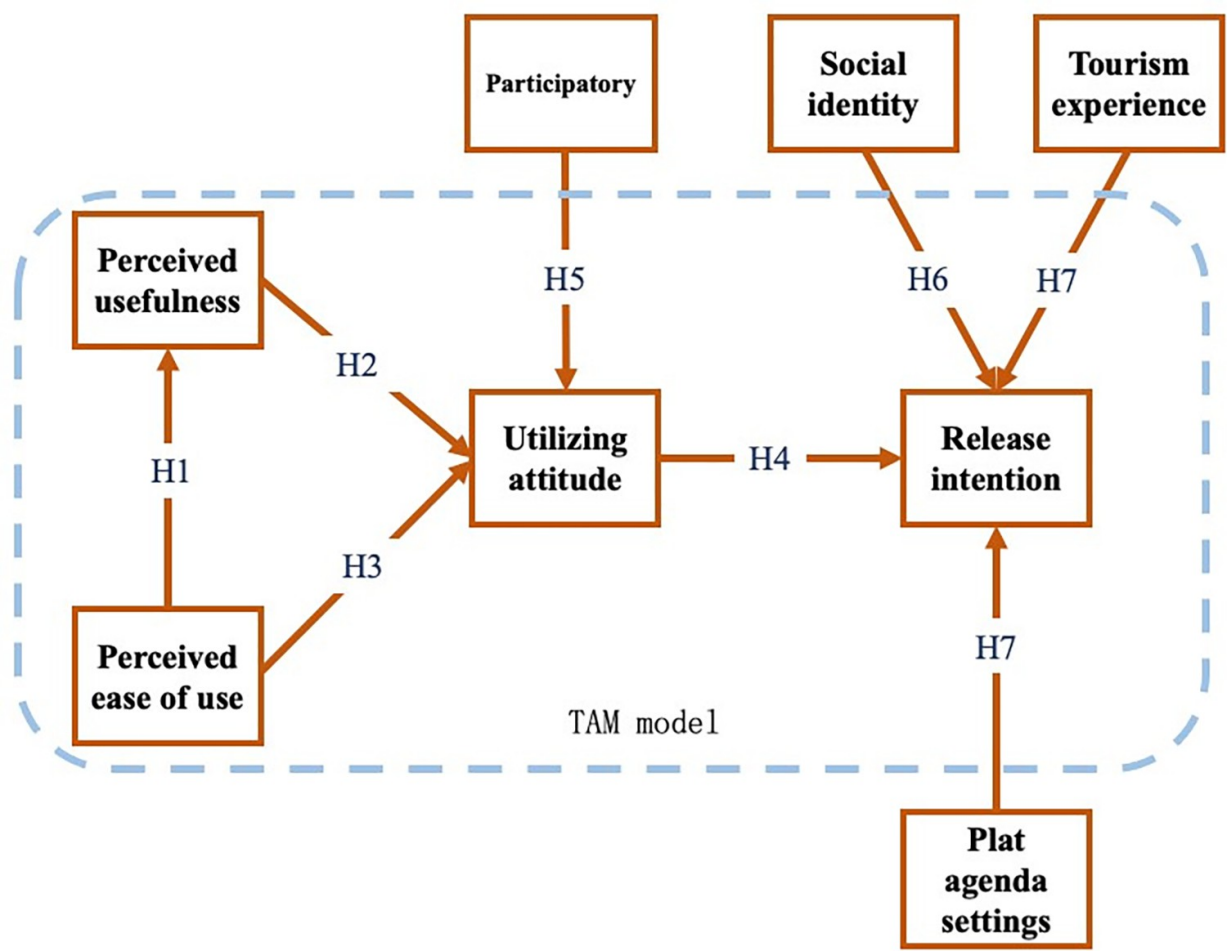
Research model.

Based on the causality diagram and technology acceptance model of users’ willingness to actively publish on tourism platforms, the motivation mechanism model of users’ willingness to actively publish on the tourism platform is drawn. The model is shown in Fig 2 (the original TAM model is shown in the dotted box).

## Study design

### Measurement items and data collection

This research focuses on tourism platform users’ psychological processes and behavioral intentions. The measures for the eight constructs were adapted from validated scales. The measures were based on five-point Likert scales, ranging from 1 (strongly disagree) to 5 (strongly agree). The perceived usefulness was measured by four items based on [53]. The perceived ease of use was measured by three items from [54,55]. The utilizing attitude was measured by three items adapted from [56]. The participatory was measured by three items adapted from [57]. The social identity was measured by three items adapted from [58–61]. The tourism experience was measured by four items adapted from [62]. The plat agenda settings was measured by three items adapted from [63]. The release intention was measured by three items adapted from [64,65].

Prior to answering the questionnaires, all participants were provided with a written informed consent and signed. The study and questionnaire design were carried out in accordance with the approval of the Ethics Committee of the Tongmyong University. The data was collected from an online survey of 552 tourism platform users residing in China, ranging from 18 to 60 years old. For data collection, we used an online panel by Dynata, are search company. The company randomly selected users between 18 and 60 who had at least one type of experience using the tourism platform from a panel in China and invited them to participate in the study.

### Descriptive analysis of demographic characteristics

SPSS 25.0 was used for the descriptive statistical analysis of the sample data. The demographics of the sample are as follows: in terms of gender, there are more female users than male users; in terms of age, users aged 35–45 years old account for the most significant number of users, 24.1%; in terms of education, the majority of users have a bachelor’s degree, accounting for 55.4%; in terms of frequency of use of tourism platform, users who use it 7–9 times a week account for the majority, accounting for 41.8%. These data indicate that the data collected by the questionnaire of this study covers a wide range of data, which is in line with the actual situation of tourism platform users, as shown in Table 2.

**Table 2:** Frequency distributions for the sociodemographic variables (N = 552).

Variables	Category	Frequency (%)	Cumulative %
Gender	Male	244 (44.2)	44.2
	Female	308 (55.8)	100
Age	Under 18	103 (18.7)	18.7
	18–25	102 (18.5)	37.1
	26–34	131 (23.7)	60.9
	35–45	133 (24.1)	85.0
	Above 45	83 (15.0)	100
Education	High school and below	88 (15.9)	15.9
	Bachelor	306 (55.4)	71.4
	Master	109 (19.7)	91.1
	Doctor	49 (8.9)	100
Experience	1–3 times	90 (16.3)	16.3
	4–6 times	165 (29.9)	46.2
	7–9 times	231 (41.8)	88.0
	More than 10 times	66 (12.0)	100.0

### Measurement model analysis

First, the reliability of the measurement model was assessed. The data results showed that the Cronbach’s alpha for all variables was greater than the critical value of 0.7 (0.701–0.872) [66], and the Composite Reliability for all variables was also greater than the critical value of 0.7 (0.831–0.921) [67]. Therefore, the measurement model has good reliability (see Table 3). Second, the convergent validity of the measurement model was assessed. The outer loadings (λ) of all question items were greater than the critical value of 0.7 (0.728–0.874) (Hair et al., 2017) and the AVE (average variance extracted) of all variables were greater than the critical value of 0.5 (0.595–0.796) (Hair et al, 2017). The measurement model had good convergent validity,as shown in Table 3. Finally, the discriminant validity of the measurement model was assessed. The square root of each variable AVE was more significant than the correlation coefficient of that variable with any other variable, and the measurement model had good discriminant validity, as shown in Table 4.

**Table 3:** Reliability and convergent validity analysis (N = 548).

Construct	Item	Factor Loading	Cronbach’s alpha	CR	AVE
Perceived usefulness (PU)	PU1	0.809	0.825	0.884	0.656
	PU2	0.814			
	PU3	0.827			
	PU4	0.791			
	PEOU1	0.818			
Perceived ease of use (PEOU)	PEOU2	0.814	0.72	0.842	0.64
	PEOU3	0.766			
Utilizing attitude (UA)	UA1	0.846	0.789	0.877	0.704
	UA2	0.858			
	UA3	0.811			
Release intention (RI)	RI1	0.881	0.872	0.921	0.796
	RI2	0.896			
	RI3	0.9			
Social identity (SI)	SI1	0.803	0.757	0.859	0.67
	SI2	0.854			
	SI3	0.797			
Participatory (P)	P1	0.764	0.701	0.831	0.622
	P2	0.754			
	P3	0.845			
Plat agenda settings (PAS)	PAS1	0.83	0.753	0.859	0.67
	PAS2	0.813			
	PAS3	0.812			
Tourism experience (TE)	TE1	0.762	0.78	0.854	0.595
	TE2	0.726			
	TE3	0.752			
	TE4	0.84			

**Table 4:** Discriminant validity analysis (N = 548).

Construct	P	PAS	PEOU	PU	RI	SI	TE	UA
P	**0.789**							
PAS	0.048	**0.818**						
PEOU	-0.014	-0.056	**0.8**					
PU	-0.069	-0.009	0.365	**0.81**				
RI	0.074	0.218	0.029	0.134	**0.892**			
SI	0.058	0.024	0.033	0.008	0.235	**0.818**		
TE	0.061	0.064	-0.031	0.071	0.237	-0.021	**0.771**	
UA	0.221	-0.017	0.183	0.383	0.237	0.032	0.051	**0.839**

Note: The diagonal elements (in bold) are the square root of variance shared between the AVEs, whereas the off-diagonal elements are correlations among constructs.

### Structural model analysis

**Fig 3:**
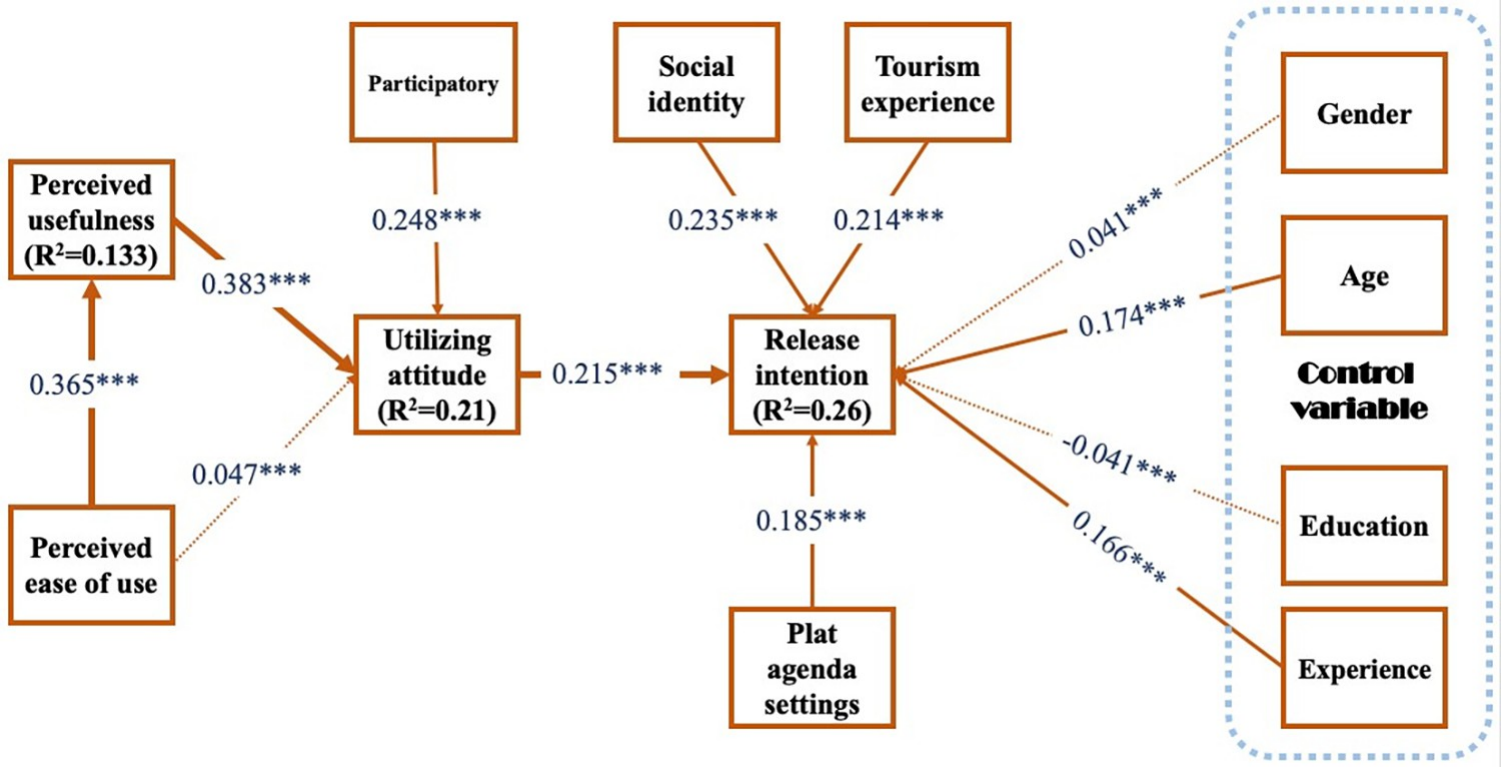
PLS results of the structural model. Note: The dotted line indicates that the relationship is not valid. ***p < 0.001; **p < 0.01; *p < 0.05.

The results of the path analysis showed in Fig 3 and Table 5: there was a positive and significant effect of PU on UA (β = 0.383, t = 8.768, p<0.001); there was a positive and significant effect of PEOU on PU (β = 0.365, t = 8.793, p<0.001); there was a positive and significant effect of P on UA (β = 0.248, t = 6.561, p< 0.001); there was a positive and significant effect of SI on RI (β = 0.235, t = 6.416, p<0.001); there was a positive and significant effect of TE on RI (β = 0.214, t = 5.66, p<0.001); there was a positive and significant effect of PAS on RI (β = 0.185, t = 4.891, p<0.001); and there was a positive and significant effect of UA on RI (β = 0.215, t = 5.421, p<0.001). Therefore, hypotheses H1, H2, H4, H5, H6, H7, and H8a establish. On the other hand, there was no significant effect of PEOU on UA (β = 0.302, t = 1.032, p>0.05). Therefore, hypothesis H3 does not valid. Regarding the control variables, Age and Experience had a significant effect on RI, and Gender and Education did not significantly effect on RI.

**Table 5:** Path coefficients and research hypothesis testing results.

	T-value	P-value	Path coefficients	Hypothesis testing results
P → UA	6.561	0.000***	0.248	H5 Establish
PAS → RI	4.891	0.000***	0.185	H7Establish
PEOU → PU	8.793	0.000***	0.365	H1Establish
PEOU → UA	1.032	0.302	0.047	H3 Not valid
PU → UA	8.768	0.000***	0.383	H2 Establish
SI → RI	6.416	0.000***	0.235	H6 Establish
TE → RI	5.66	0.000***	0.214	H8 Establish
UA → RI	5.421	0.000***	0.215	H4 Establish
Gender → RI	1.111	0.267	0.041	/
Age → RI	4.59	0.000***	0.174	/
Education → RI	1.049	0.294	-0.041	/
Experience → RI	4.32	0.000***	0.166	/

***p < 0.001; **p < 0.01; *p < 0.05.

## Discussion and conclusions

### Key findings

First, perceived ease of use has a significant effect on perceived usefulness. This indicates that users find it easy to use the eWOM feature of the tourism platform. At the same time, this feature offers reference value for users to choose travel destinations [21], which reduced users’ perceived opportunity cost [68], especially when traveling extensively. At the same time, browsing other users’ eWOM also creates perceived usefulness regarding their travel destination selection [6]. Further, perceived usefulness has a significant effect on usage attitudes. This suggests that users change their attitude toward publishing eWOM on the tourism platform after getting their target value. Users will adjust their emotions and be more proactive in publishing eWOMs afterwards. Also, usage attitude significantly affects release intention, which shows that users’ willingness to actively publish eWOM increases whilest usage attitude increases. Participatory has a significant effect on utilizing attitude, which indicates that users perceive the platform as unrestricted and offering a low threshold for creation with decentralized characteristics. The qualities of the tourism platform will correspondingly increase users’ willingness to actively publish eWOM.

Second, perceived ease of use on utilizing attitude was not significant. This indicates that the related usage features of the tourism platform do not significantly affect users’ willingness to actively publish eWOM. The reason may be that the features of the popular tourism platforms have all matured during past product iterations. They have been gradually converging, and usage turns out to be very convenient. In this survey, 83.7% of users use the tourism platform at least four times a week. Most users have mastered the basic operations of publishing eWOM. Therefore, the simplicity of the eWOM function does not affect their attitude towards using it. While functionality and feature set is more and more diversified, platform complexity is also increasing. It takes only a few seconds for a user to publish an eWOM. However, when the user posts an eWOM with a video, it takes more time to add filters, effects, and music to their videos. As a result, users think that the steps for publishing eWOM on the platform need not be overly cumbersome. Users are not willing to invest too much time. They only need to use the primary text and image functions to publish eWOM about the destination. Thus, perceived ease of use does not motivate tourism platform users’ willingness to actively publish eWOM. To analyze the findings in more depth and nuance, we conducted in-depth interviews with a diverse group of experienced tourism platform users, including frequent and occasional users, who provided valuable insights into their motivations and experiences with eWOM. During the interviews, users consistently expressed that ease of use played a minor role in their utilizing attitude. They reported being much more interested in the tourism platform’s content and the community composition rather than focusing on features related to ease of use, such as interface design and interactions. They emphasized that most tourism platforms offer welcome tutorials, help centers, or customer support, facilitating their learning of basic usage and resolving any encountered issues. Furthermore, users generally consider most tourism platforms’ design and user interface intuitive and user-friendly. Our interviewees also shared that they were more motivated to actively publish eWOM by rewards or incentives offered by the tourism platform, social pressure, and alignment with their values. This finding reinforced our quantitative results, which indicated that users needed to be more sensitive to ease of use when actively publishing eWOM on tourism platforms. By integrating these qualitative insights, we hope to provide a more comprehensive understanding of user motivations and the factors driving their willingness to actively publish travel-related eWOM on tourism platforms.

Third, social identity, tourism experience, and platform agenda setting significantly affect release intention. First, it indicates that users want to gain recognition from others when publishing eWOM on the platform [69]. On the one hand, users seek visibility by publishing eWOM, hoping to gain online status symbol and reputation [69]. On the other hand, users want to seek a sense of belonging and accomplishment, desiring for a sense of dependence from the group they belong to and affirm their identity value [70]. Second, the tourism experience shows that when users travel on the ground in a tourist destination, the scenery of the destination inspires nostalgia, homesickness, and other emotions. At the same time, travel destinations also remind users of their friends and relatives, traditional culture, or historical events in their hometown, etc. These nostalgia feelings will stimulate users’ emotions and generate motivation to publish eWOM on the platform [71,72]. Finally, the platform agenda setting indicates that the platform’s prize participation topics and current hot eWOMs will inspire users to publish eWOMs on the platform [73]. At the same time, topics with emotions can inspire users to create ideas and increase their willingness to actively publish.

Fourth, for the control variables, we find that users who frequently use the tourism platform or are older are more likely to actively publish eWOM on the platform. In the context of the tourism platform, middle-aged and older users have more time to use the tourism platform and browse travel information. As a result, they are more willing to spend time publishing eWOM on the platform about the tourist destinations they have visited. Similarly, frequent users of the tourism platform are more aware of the platform’s rules or rewards available, such as publishing eWOM to participate in point sweepstakes. These factors will also stimulate them to actively publish eWOM on the platform.

### Theoretical contributions

First, this study address previous research’s lack of discussion on the motivational mechanism of consumers’ proactive publishing intention [68,74,75]. We propose a model of the motivational mechanism of users’ willingness to actively publish on tourism platforms with system dynamics and the technology acceptance model combined, which is the first and is crucial for the future development of dynamic thinking in the study of user intention within the specific context of tourism.

Second, this study offers innovative variable inspection in the dynamics of users’ willingness to actively publish on the tourism platform. With users’ willingness to actively publish on the platform as the dependent variable, different streams of independent variables are included in terms of characteristics (perceived usefulness, perceived ease of use, plat agenda settings), users (participatory, social identity), and tourism destinations (tourism experience). Further, the specific matching relationship between tourism platforms’ characteristics and users’ eWOM motivations is studies. Overall, this study explores users’ choice and adoption of tourism platforms from multiple dimensions, which also extends the research stream related to technology adoption [76–79].

Third, our research methodology is innovative with the use of system dynamics, TAM, survey, and structural equation modeling. Most previous studies on eWOM on tourism platforms were based on a single research method, such as textual analysis and qualitative analysis [80,81]. Few studies have combined different research methods, which has the advantage of making up for the deficiencies in a single method. The multiple research methods to investigate users’ willingness to actively publish on the tourism platform would provide a reference for subsequent research.

### Practical contributions

This study implies practical advice for platform operators. First, platform operators may benefit from strengthening the quality management of eWOM content, which can be examined comprehensively in terms of richness of content, the vividness of language, and clarity of expression. For high-quality eWOM, operators can add a tag such as "Excellent eWOM." This will help users filter out useful eWOM faster and increase the reach of quality eWOM. Secondly, to increase users enthusiasm to publish quality eWOMs, operators can launch a quality eWOM competition. By weighing the number of likes, comments, and shares of each eWOM, the heat value of the eWOM could be calculated. Then the content could be ranked and rated according to its heat value. At the same time, the operator can set up points rewards, travel product rewards, etc., as appropriate. Second, establish a perfect evaluation system of the publisher’s professionalism. Users can be rewarded with reward tokens for each additional tourism experience, publishing an eWOM, and likes from others, replying or forwarding eWOMs, etc. Based on accumulated tokens, the operators classify the publishers into different professional levels. The publisher’s professional rank and gold coins will be made public. Implementing the reward model may fully mobilize enthusiasm for user interaction. At the same time, users will give gold coins to their friends on the platform, enhancing the strength of the user relationship chain. Third, create an atmosphere where users publish eWOM on the platform.

This study also implies advice for tourist destinations. First, strengthen the cooperation with tourism platforms. Tourism destination managers need to pay attention to eWOM and explore its further utility. Quality eWOM can be used as marketing material. Negative eWOM can be used to urge tourism managers and service providers to improve their services. Tourism destinations can cooperate with the platform to carry out eWOM awards and travel photography contests. The activities will motivate more users to actively publish eWOMs and increase the visibility and influence of the tourism destination. Additionally, tourist destinations can invite professional publishers from the platform to visit their local areas. Do encourage them to publish professional and objective eWOM. Second, destination managers should refer to the eWOM posted by users on the platform. According to their feedback to improve tourist places’ facilities, environment, services, etc. Thus, creating a better tourism experience for users. Third, tourism destination managers should adopt differentiated marketing for different user groups to meet the needs of different groups. This will achieve higher user satisfaction and promote the spread of relevant positive eWOM.

### Limitations and future research

The questionnaire method is susceptible to the subjective influence of the respondents, such as time, space, and other contextual variables. The reliability and trustworthiness may not be particularly strong. The research results obtained are not suitable for too much inference. A more objective and systematic study through behavioral experiments or grounded theory is expected in the future. Second, the survey sample of this study is based on Chinese users. Future studies can sample other cultural and subcultural groups in survey research, such as Generation Z, college students, users in other countries, etc., to gradually form a complete theoretical framework.

## Supporting information

S1 Data (TXT)
